# Synopsis of Proceedings Texas State Med. Ass’n.

**Published:** 1891-05

**Authors:** 


DANIEL'S

### Texas Medical Journal.

A Representative Organ of the Medical Profession, and an Exponent of Rational Medicine; devoted to the Organization, Advancement and Elevation of the Profession in Texas.

PUBLISHED MONTHLY.—SUBSCRIPTION $2.00 A YEAR.

Vol. VI. AUSTIN, MAY, 1891. No. 11.

### Society Notes.

### TEXAS STATE MEDICAL ASSOCIATION.

### Synopsis of Proceedings of the Twenty-third Annual Session.

FIRST DAY—MORNING SESSION.

WACO, TEXAS, April 28, 1891.

THE TEXAS STATE MEDICAL ASSOCIATION met in annual session at City Hall, in Waco, Tuesday, April 28, at 10 a. m. Meeting called to order by Dr. J. H. Sears, chairman of committee of arrangements. After prayer, Hon. C. C. McCulloch, Mayor, delivered an address of welcome, and Dr. W. H. Wilkes was introduced. He welcomed the Association in behalf of the local profession. His speech sparkled with flashes of real wit; he extended welcome, and offered the Association all the cold water they needed or might want, but hoped nobody would throw any on our meetings; spoke of the products of Waco, and cited her contribution of Coke as a big thing, etc. But at subsequent hours the guests were offered something besides cold water. He had been accused of "guying," but he was no guy–sir, the geyser was on top of the hill, etc.

After Dr. Wilkes, came Mr. Flint, a lawyer of Waco, who delivered an address also, full of good wishes.

Chairman Sears them made his report as chairman of the committee of arrangements, and extended many invitations to the members to go to places, and the invitations, he said, included the ladies, of course, who had accompanied delegates. Among the invitations were those to several receptions, one at the residence of Hon. George Clark, at residence of Colonel Prather, and some others not remembered, and a banquet and reception at the McLennan Hotel in especial honor of the guests. All these social features were brilliant, and much enjoyed.

The President, Dr. W. P. Burts, was then escorted to the chair, and responded briefly to the several addresses of welcome. He then declared the executive morning session open for business, and read his annual message and recommendations. It was referred to a committee of which Dr. J. D. Osborn was chairman. The committee reported later, advising the adoption of the several suggestions made by the President for the good of the order. Report received, and committee discharged.

Roll call and reading of minutes of last meeting being dispensed with, on motion, reports of officers and committees were in order.

The Secretary read his report, and his financial report. The latter is given, for the benefit of those who paid dues to him instead of to the Treasurer, as they should have done. The Secretary's report was referred to a special committee who, having examined it, reported it all right, and recommended its adoption. Report rceived, and committee discharged.

SECRETARY'S FINANCIAL REPORT.

1890–1.

Debits —Cash received since last meeting, as follows: W. H. Lancaster, T. M. Stone, J. F. Dean, G. M. Stevens, S. H. Barham, J. R. Reeves, W. R. Vaughn, J. H. McCaleb, Jno. Preston, A. D. Paulus, G. M. D. Patterson, R. C. Nettles, J. A. Matthews, J. M. Dollar, A. N. Denton, S. W. Sholars, R. Atkinson,

G. W. Kerr, J. W. Miller, F. H. Tucker, W. Mayers, R. T. Knox, C. McManus, E. W. Link, I. E. Clarke, J. V. Spring, J. C. Jones, D. Parker, A. S. Wolff, S. Eagan, J. Braunagel, W. T. Jones, J. F. Y. Paine, J. M. Fly.—34 at $5 each, $170.

H. F. Cooke, F. S. White, J. F. Starley, R. P. Talley, J. H. Ferris, G. W. Christian, S. L. Gilbert, E. L. Sessions, C. S. Bubo, F. C. Ford, T. S. Burke, P. Jordan, F. Paschall, F. Gray, E. J. Beall, R. Steiner, J. A. White, N. B. Shands, W. M. Burger (with application, April, 91).—19 at $10 each, $190.

D. R. Wallace, $20; J. O. Lewright, $15; N. A. Olive, $15; J. M. Burleson, $8.50; two copies Transactions, $2.—$60.50.

| | | |
|---|---|---|
| Total debits . . . . . . . . . . . . . . . . | | $420 50 |
| 1890–1. | | |
| By disbursements as per vouchers 1 to 11 . . | $114 93 | |
| Balance turned over to Treasurer, receipt with vouchers . . . . . . . . . . . . . . . | 305 57 | |
| Total . . . . . . . . . . . . . . . . . . | | $420 50 |

E. & O. E. Austin, Texas, April 27, 1891.

F. E. Daniel, M. D., Secretary.

Referred to committee, who later approved same as correct.

The Treasurer then read his yearly report, which, on motion, was also referred to a committee, and reported correct.

The publishing committee then made their report, showing the work done by them, including the issue of 650 copies of Transactions, in cloth, at a cost of about 83 cents each, and the work done for the committee on legislation. The committee showed that in consequence of the policy of withholding the Transactions from those who do not pay up promptly, there were on hand 148 copies of 1890, and in all, as far back as 1884, 311 copies. The chairman (Secretary) suggested that these surplus copies be donated to the libraries of the several local societies, and the committee appointed to examine report so recommended.

[They have been turned over to Dr. West, Secretary elect, and societies wishing a copy of each or either year must apply to him.—Ed.]

The committee on medical legislation made no report. Committee on collection of surgical cases made no report.

The Judicial Council was called, and of the regular members only Drs. T. J. Bell, I. E. Clarke, J. V. Spring, J. C. J. King and H. L. Fountain were present. Dr. King being First Vice-President, was excused, and Dr. A. C. Walker was appointed in his place. The President appointed to the vacancies Drs. S. R. Burroughs, A. B. Gardner, of Beellville, J. C. Loggins, R. E. Moody, S. W. Sholars, W. Bev. West, W. T. Evans.

Judicial Council retired for work, and organized with Dr. Bell chairman, and Dr. Loggins secretary.

The memorial session was appointed for 11 a. m. Thursday.

The Address in Medicine was then read by Dr. H. A. West, Chairman of Section on Practice, which, on motion, was referred to the publishing committee. Adjourned to 2 p. m.

---

AFTERNOON SESSION.

The Section on Practice of Medicine, Materia Medica and Therapeutics was called, and the President, Dr. Burts, resigned the chair to Chairman Dr. West; Dr. Church, the Secretary, being absent, Dr. R. W. Knox was appointed Secretary of Section.

Dr. C. B. Raines, of Mineral Wells, read a paper on the plague at Aurora, Wise county, written by Dr. E. J. Beall, of Fort Worth. In this able paper it was recited that January 20, 1890, James Jefferson and his mother arrived at Aurora from Indian Territory, blind and without toes, the result of a disease contracted in the Indian Territory. The mother died and the disease spread, getting into the Deerberry house. Several died. After denying that it was typhus or cerebro-spinal fever, and giving interesting reason for such denial, Dr. Beall concluded that it was hematytic spinal fever with peri and intra-articular inflammation, a plague which appeared ninety-five years ago in the State of Massachusetts, and never reappeared until last year, at Aurora. It was called by the Massachusetts doctors "spotted fever."

Dr. Beall's paper was discussed and referred to the committee on publication.

Dr. W. A. Morris, of Austin, presented a paper on the effect of alcohol, taking strong ground against its use, admitting that it has its place, but restricting that space to very narrow limits.

Dr. Morris' strictures were particularly striking on the point of transmission hereditarily, and the effect of stimulants on children.

Drs. Dudley, of Hillsboro; Sears, of Waco; Wallace, of Terrell, and several other physicians, spoke in highest terms of Dr. Morris' paper, after which it was referred to the publishing committee, with instructions to publish it. This is one of the ablest papers read in the Association, and a copy of it should be in every house in America; it was almost prophetic, and came with double force from an octogenarian in hale health who is a living exemplification of his views.

Dr. J. W. Carhart, of Lampasas, read a paper on substitution of cheap pharmaceuticals in the filling of prescriptions. Among abuses recited by Dr. Carhart was the refilling of prescriptions without reference to the physician writing it, and frequently in defiance of his protest. This, and the evil referred to in the title of the paper appears to be disagreeably prevalent, at least in some localities, with prescriptionists.

Section on Practice closed.

Section on Surgery was called.

In the absence of Dr. S. L. Gilbert, of Sulphur Springs, Chairman of the Section on Surgery, Dr. C. H. Wilkinson, of Galveston, was appointed in his place.

In this Section Dr. B. E. Hadra, of Galveston, read a paper on "The Wiring of the Vertebræ as a Means of Immobilization in Fracture and Potts' Disease."

The reading of Dr. Hadra's paper was followed by discussion, in which Dr. Fontain offered a plan of sawing and dovetailing, to gain the end sought, in preference to wiring.

Dr. Hadra's paper was received and referred to the publishing committee.

A patient was offered by Dr. C. F. Paine, of Comanche, to illustrate a case of "Chronic Suppurative Inflammation of the Elbow Joint."

After the discussion of this case the convention adjourned until Wednesday morning.

Tuesday afternoon, from adjournment at 4:30, was spent in riding over the beautiful city, under escort of the committee of local physicians, visiting the famous spouting artesian wells and other points of interest, and in the evening the members attended a lecture by Henry Watterson.

---

SECOND DAY—MORNING SESSION.

WEDNESDAY, April 29, 1891.

The Association was called to order at 9 a. m., by President Burts. Prayer. Minutes of the first day read, and after correction of a minor error as to memorial service hour, were adopted.

Dr. J. M. Meyer, of Danville, Ky., was introduced by Dr. Knox, and on motion, Dr. Meyer was unanimously accorded the privileges of the floor.

Section on Surgery recalled. Dr. C. H. Wilkinson, chairman pro tem., took the chair.

Dr. B. E. Horton, an applicant for membership, was introduced and read a paper on Cerebral Localization, which, on motion, was referred to the publishing committee.

Dr. J. J. Burroughs then read the account of the case of Cæsarean section mentioned in this journal for April. The operation was performed on account of congenital deformity of pelvis and was a creditable success. Dr. Burroughs exhibited photos of patient after recovery, showing seat of incision, the baby, etc. This paper elicited much interest and spirited discussion. Paper was referred.

Section on Surgery closed and Vice-President Dr. J. C. J. King took the chair.

Dr. J. R. Briggs read a telegram from the Pharmaceutical Association in session at Dallas.

He also offered the following resolution, and made a speech in support of it:

WHEREAS, Strong efforts will be made at the next session of the American Medical Association by certain physicians of the East to secure the removal of the Journal of the American Medical Association from Chicago to Washington, D. C., therefore,

*Resolved*, That the Texas State Medical Association instructs its delegates to the National Association to vote against such removal, first, last and all the time.

The resolution was tabled by a strong majority.

A letter was read from Dr. George J. Engelman, president of the Southern Surgical and Gynecological Association regretting enforced absence and expressing high regard for the Texas State Medical Association.

Dr. J. M. Autrey, of Houston, and D. C. Hewson, of Orange, resigned by letter. Accepted.

Rev. Dr. Burleson, president of Baylor University, addressed the Association and invited them to visit the university. Invitation received with a vote of thanks. Adjourned.

In the afternoon in the absence of Dr. Burts, Vice-President Reeves called the Association to order.

The Judicial Council reported as follows:

The Judicial Council would report that we have examined the credentials of the following delegates and find them entitled to seats in the Texas State Medical Association.

Austin district—Dr. J. W. Carhart, J. A. Halloway and J. A. Davis.

Austin District Medical Association—H. H. Thorpe.

Austin District Medical Association—J. T. Barnwell.

Central Texas Medical Association—Drs. R. C. Nettles, R. F. Minnock and W. C. Blalock.

Central Texas Medical Association—Drs. H. L. Foster and J. C. J. King.

Central Texas Medical Association—Drs. A. H. Snead, W. W. Wilhe, J. M. Jordon, W. R. Blailock, W. E. Menefee, R. E. Thompson, S. V. Morgan, H. M. Stuart, J. D. Law, M. D. Knox, S. D. Davidson, J. C. Shaw, Douglass Harris, J. H. Witte, W. B. Carpenter and J. T. Valeant.

Smith County Medical Association— C. A. Smith, A. G. Baldwin and A. L. Montgomery.

Hill County Medical and Surgical Society—J. W. Miller.

West Texas Medical Association—Dr. J. V. Spring.

West Texas Medical Association—Dr. James Kennedy.

Galveston Medical Association—C. H. Wilkinson, A. Withy and R. C. Hodges.

At this stage the Judicial Council entered and reported the following named delegates, regular, entitling them to seats:

Drs. G. W. Christian, T. D. Wooten and T. J. Bennett, Austin district.

Dr. George Cupples, West Texas Association.

Dr. B. F. Church, Terrell Medical Association.

Dr. W. T. Richmond, Austin District.

Section on Obstetrics called.

In the Section on Obstetrics and Diseases of Children, (the chief work of the afternoon session), Dr. J. W. Carhart, of Lampasas, presided.

The Secretary of this Section, Dr. J. H. McCaleb, of Webberville, being absent Dr. J. A. Holloway, of Round Rock, was elected to fill his place.

Dr. J. F. Y. Paine, of Galveston, read a paper entitled, "Some Practical Observations on the Management of Albuminuria in Pregnancy."

This paper provoked discussion participated in by Drs. T. D. Wooten, Wharton, Pitts and others.

It was referred to the committee with orders to publish.

Dr. T. J. Bennett, of Austin, followed, on "Prevention of After Pains." This was discussed by Dr. Paine, and referred to the committee on publishing.

In conclusion of the afternoon session of the second day Judge J. C. Walker read a paper on electricity. This was outside the programme and the reading was in accordance with a vote to that effect.

Judge Walker's paper was lengthy, highly scientific and was listened to with profound attention.

Dr. Daniel addressed a letter to the Judicial Council, calling their attention to an article in the February number of the *Texas Health Journal*, in which his moral and professional character had been assailed by Dr. J. R. Briggs, and respectfully asked an investigation of the same, submitting documentary evidence to disprove the allegations. One of the charges was that he, Dr. Daniel, had "left Mississippi under a cloud," and settling in Denison, flooded the town with advertising handbills. Amongst the evidences submitted, was an official statement from the secretary of the Mississippi Medical Association, with the seal of the Association affixed, and letters from citizens of Mississippi.*

He was never an hour in Denison in his life. Many of the allegations in the *Health Journal*, were disproved by documents in Dr. Briggs' own handwriting.

The Judicial Council then reported as follows: "We, the Judical Council, having carefully examined the charges against Dr. F. E. Daniel by Dr. J. R. Briggs, published in the *Texas Health Journal* for February, 1891, hereby exonerate Dr. Daniel from any and all charges of conduct unbecoming a gentleman and physician and a member of this Association, and expel Dr. J. R. Briggs from this Association.

BELL, Chairman,
LOGGINS, Secretary.

On motion, the report of the Judicial Council, was received and adopted.

Dr. Daniel then rose, and after reviewing his labors in behalf of the Association, and stating that he had served as Secretary on the death of Dr. Burt, by especial request of the President, at the instigation of a number of members; that he had been unanimously elected in 1887, for five years; that at the end of three years he had resigned, because of some disatisfaction on the part of certain members, whom he had offended through the JOURNAL;

---

*One from the Chief Justice of Supreme Court; the Secretary of State; the Grand Secretary of the Grand Lodge of Mississippi; the President of the Howard Association, etc., testifying to the high character and standing of Dr. Daniel in Mississippi, as a gentleman and a physician; to his services in yellow fever epidemics, etc.; and also a document from the Secretary of the Mississippi State Medical Association, under seal of the society, which reads as follows:

Mississippi State Medical Association,
Office of Secretary,
JACKSON, MISS., April 16, 1891.

TO WHOM IT MAY CONCERN:

This is to certify, that Dr. F. E. Daniel, was for many years a highly honored member of this Association, held in the highest esteem, on account of his great medical education, and his uniformly gentlemanly deportment. As an evidence of the esteem in which he was held, I will state that at the Winona meeting, in 1881, he was unanimously chosen to deliver the annual oration for this body. That Dr. F. E. Daniel was ever "under a cloud" in Mississippi, has no foundation whatever in fact, and was totally unknown either to the medical profession, or the citizens of Mississippi.

Signed, W. E. TODD, M. D.,
Secretary Miss. State Med. Ass'n.

[Seal.]

he had resigned in the interest of harmony, whereupon the majority had immediately re-elected him. He had now served one year more; and as certain members did not accord to a paid officer the right to criticise any act of the Association, or of members, through the JOURNAL, and as he felt it his duty to do so sometimes in the interest of the Association, he was not willing to be thus hampered; and that no one, however prejudiced, should truthfully say that he was an element of discord, or an impediment to progress, he must insist on his resignation being again accepted, and finally; he could not consent to serve another day.

It was understood that this was in the interest of peace, and that not a word of complaint had ever been heard as to his official work.

Dr. Daniel's resignation was accepted, and Dr. H. A. West, was appointed Secretary pro tem.

On motion the Association adjourned.

---

THIRD DAY—MORNING SESSION.

THURSDAY, APRIL 30, 1891.

President Burts in the chair. 9 a. m.; prayer.

Reading minutes dispensed with on account of temporary absence of ex-Secretary. Dr. West was installed as Secretary, having been appointed by the President for the unexpired term of four years of Dr. Daniel's term, and delivered an address. He had thrown himself into the breech [like that fellow of old, whose name we have forgotten] and so forth.

Nominating committee was organized and requested to meet at 1 p. m.

Section on Obstetrics, having adjourned yesterday before completing its work, was called. Dr. Carhart, chairman, taking the chair. He read a paper entitled: "A Step Backwards, or the Legal Control of Marriage." He declared in this paper that "the Malthusian idea as to the limitation of increase of population seems now to be fully operative, so far as the better classes are concerned." He suggested restriction of issuance of licenses and a medical board to pass upon the physical qualifications of persons seeking matrimonial happiness. Paper received and referred.

The hour for memorial service having arrived,—11 a. m.—it began with an address by Dr. Carhart, chairman of committee on necrology; most appropriate, and feelingly delivered. The members deceased since last meeting are: Drs. S. A. Towsey, Galveston; J. H. T. King, Laredo; J. H. Smith, Dallas; A. A. Terhune, Jefferson; L. J. Graham, Henderson; F. T. Paine, Comanche; G. W. Kerr, Waelder.

Dr. W. A. Morris and several other members spoke in feeling terms of the deceased members.

Bussiness being resumed, the Section on Gynecology was called, and Dr. C. F. Paine, chairman of Section, read his address. It was a general report on the advances of gynecology. Paper received and referred as usual.

A paper by Dr. O. Eastland, on Pemphigus, was read by title and referred.

Adjourned to 2 p. m.

---

AFTERNOON SESSION.

Meeting called to order; several papers were read by title and referred.

Dr. B. F. Brittain read a paper entitled: "Is Small-pox Contagious During the Initial Fever?"

The reasonings, illustrations, examples and cases quoted, went to show that it was only in the advance stage that it is contagious. The Doctor drew his instances chiefly from the recent plague which afflicted some Texas towns severely and even got into Waco. It is all gone now and the doctors are availing themselves of the only thing of value about it, namely, the experience.

In the stage of secondary fever, when the odor is intense, then the microbe is most active and liable to infect people coming within the radius of their leap.

That appeared to be the generally accepted opinion.

Dr. J. A. Wysong, of Galveston, read a lengthy paper on Clinical Examination in Kidney Troubles, which was referred to the committee on publication, after liberal discussion in which Dr. Wysong's paper was greatly praised.

Dr. Pitts, of Washington county, took exceptions at remarks of Dr. Ward, that examinations for insurance were slightingly made by physicians, and expressed himself warmly in the matter.

Dr. Ward elaborated his first remarks, making himself clearer, and this brought about a satisfactory explanation and settlement.

Dr. Noyes, of Detroit, took part in the discussion, and spoke highly of the paper.

Dr. Dudley, of Hillsboro, added his experience.

Charges brought by Dr. J. D. Burch, against Dr. F. E. Daniel were dismissed as unsupported by any evidence.*

The Judicial Council reported also that Dr. Ghent was exonorated from charges of conduct unbecoming a gentleman and physician—no such charge having been made, so far as we were able to learn—*supposed* to have reference to the sale of hepatic pills, attention to which had been called in the JOURNAL last December.

A resolution to reconsider the action of the Council expelling Dr. J. R. Briggs, was tabled.

In conclusion the Judicial Council submitted a recommendation that for all future time doctors settle their personal differences outside of the Association.

The entire report was adopted.

The nominating committee reported the following new officers to serve for the ensuing year:

---

*The charge brought by Dr. Ghent through Burch was "malice" in denouncing the sale of Hepatic Pills, and "tampering with a member of the Judicial Council,"—use having been made of one of our confidential letters to a gentleman, who confided in a supposed friend, and was betrayed. His 'friend' carried the contents to Burch without the knowledge of the owner of the letter, and he—used—it—for—this. Burch, Ghent's man Friday—who, for no rerson that we can see, except to play sycophant to the great unterrified proprietor of Ghent's Hepatic Pills—whom he didn't know—wrote himself an ass, and said we deserved to be munched, and that it took just such a kingly muncher to munch us, as the said unterrified "lion"—[ugh]—Burch is now on the Judicial Council, and his munching lion may seek whom else he may munch in his "kingly" manner, and lick his chops in peace; thus merit hath its own reward.—ED.

President, Dr. W. H. Wilkes, of Waco.

Vice-President, Dr. P. C. Coleman, of Colorado.

Second Vice-President, Dr. W. L. Rodgers, of Temple.

Third Vice-President, Dr. B. H. Vaughn, of Hill county.

Secretary, Dr. H. A. West, of Galveston.

Judicial Council, Drs. J. D. Burch, of Aurora; J. C. Loggins, Ennis; C. P. Hudson, Alvarado; O. I. Halbert, Waco.

Dr. W. H. Wilkes was led forward and introduced by Dr. J. F. Y. Paine, of Galveston.

Dr. Paine's graceful introducticn was followed by Dr. Wilkes' address of acceptance.

This was followed by Dr. Burts, the retiring President's valedictory.

Both these addresses were well delivered and were greeted with rounds of applause.

Dr. P. C. Coleman and the other vice-presidents were led forward by committee and each installed in office, each making pleasant remarks.

In this way all the new officers were installed.

The Section of Ophthalmology and Otology was now opened, and Dr. R. C. Hodges read an interesting paper in this section, which was discussed, after which the paper was referred to the committee on publication.

A communication was read offering the doctors an excursion to Mexico for one fare over the Missouri, Kansas and Texas.

Thanks of the Association were tendered Judge J. C. Walker for his able paper on electrity, and the paper was referred to the committee on publication.

Dr. W. A. Howard presided in the Section of Surgery in the afternoon.

The Section on Medicine, Materia Medica and Pathology was opened, Dr. A. M. Douglass presiding.

Dr. J. L. Cunningham, of Fort Worth read a paper on Lacto Metrical Test of Milk. The Doctor had investigated his subject very thoroughly.

On favorable report of the Judicial Council, the following new members were admitted:

Drs. W. M. Burger, Wm. John Matthews, John E. Morris, W.

H. Seale, W. B. Carpenter, R. S. Sims, T. B. Taylor, R. W. McGhee, J. T. Carter, W. T. Richmond, Geo. W. Larendon, J. T. Barnwell, J. S. Poyner, J. C. Jarrett and James M. Pratt.

Drs. R. T. Pope, R. E. Houghton, D. W. Montgomery, W. D. Wilkes, W. P. Means, G. B. Forene, James Kennedy, C. T. Crow, Melton M. Scott, W. C. Jones, J. H. Wysong, D. B. McGhee, L. L. Jones, Thomas A. Pope, P. K. Wortham, Wm. T. Blewitt, A. P. Baldwin and J. J. Fouts.

The Council reported for membership: Drs. Wm. M. Cunningham, G. T. Thomas, T. A. Miller, J. N. Tuttle, J. N. Chandler, B. A. Fowler, and John R. Frazier.

Drs. M. D. Knox, of Hillsboro; R. C. Brown, F. W. Burger, and W. O. Wilkes, of Waco, were recommended and received as members.

The Council also recommended as honorary members the following gentlemen:

Drs. Hunter McGuire, of Richmond, Va., and J. M. Meyer, of Danville, Ky.

---

### FOURTH DAY.

We failed to get any notes of what was done Friday morning, —very little of importance.

Time and place of meeting: Tyler, 4th Tuesday in April, 1892.

Thursday evening Dr. Burts, the retiring President, delivered his address to a large audience of ladies and gentlemen. His subject was "Life." He treated it from an evolution standpoint,—narrowed it down to the existence of a "vital principle" co-existent with all matter—a principle given it by the Creator, and which could not be analyzed, nor even appreciated, except by its manifestation. No science could exactly define its connection with matter. He also recorded his belief that there can be no clash between religion and science, that in the end they must confirm each other,—religion being the word of God, and the laws which govern the universe, and science being the interpretation of that word.

It is to be regretted the papers did not publish the address. It will appear in the Transactions.

Adjourned to April, 1892.

